# Are questionable research practices considered a successful career strategy? A novel implementation of the implicit association test

**DOI:** 10.1007/s11192-025-05357-4

**Published:** 2025-07-05

**Authors:** Antonia Velicu, Fabian Winter, Justus Rathmann, Heiko Rauhut

**Affiliations:** Department of Sociology, University of Zurich, Andreasstrasse 15, Zurich, 8050 Zurich Switzerland

**Keywords:** Survey research, Scientific misconduct, Questionable research practices, Implicit association test

## Abstract

Scientific misconduct and questionable research practices (QRPs) pose significant challenges to the integrity of academic research. This study therefore investigates scientists’ implicit associations regarding misconduct and its relationship with perceived academic success. Employing the Single-Category Implicit Association Test (SC-IAT), the attitudes of 11,747 scientists across Austria, Germany, and Switzerland were examined. Results suggest that only a very low fraction of researchers associate serious misconduct with success, yet approximately one-fifth of participants associate QRPs with success. Gender and discipline-based distinctions were minimal, with notable inclinations among PhD students decreasing with status. However, limitations exist, including the lack of validation against explicit attitudes. The decline in the association between QRPs and success with status raises questions about cohort effects or shifting ethical norms. Early intervention and ongoing training efforts may mitigate these issues, emphasizing ethical considerations in scientific practice from undergraduate education onwards. Understanding these dynamics is crucial for fostering integrity in research and guiding future interventions.

**Supplementary information:**

The online version contains supplementary material available at 10.1007/s11192-025-05357-4.

## Introduction

Several scandals of scientific misconduct and questionable research practices have rattled academia in the past, and the most prominent ones have created attention way beyond the field of science (Hu, 2016). The US-governmental Office of Research Integrity (ORI) defines research misconduct as “fabrication, falsification, or plagiarism in proposing, performing, or reviewing research, or in reporting research results” (The Office of Research Integrity, 2018), often abbreviated as FFP.^1^ In addition to these modes of relatively clear scientific misconduct, there are further questionable research practices (QRP) that cannot easily be classified as right or wrong. Therefore, these are not seen as misconduct in all their manifestations and by the scientific community. This includes above all ethical misconduct in the research process (Banks et al., 2016).

Several studies seek to estimate the prevalence of the “mortal sins” (Falsification, Fabrication, and Plagiarism; FFP) and of the questionable research practices (QRP) in science (Fanelli, 2009; Pupovac & Fanelli, 2015). Meta analyses find 1.97% of scientists to admit to data fabrication and falsification (Fanelli, 2009), 33.7% to admit QRP (ibid.), 1.7% to admit plagiarism (Pupovac & Fanelli, 2015) in their own work.

Although there is rich literature on the frequency of FFP and QRP, literature on attitudes towards these behaviors is quite scarce (Tang & Chamberlain, 1997; Okonta & Rossouw, 2014). The available literature typically covers selected disciplines of the natural sciences (Pupovac et al., 2010; Holm & Hofmann, 2018), mostly (bio-)medicine, and often only investigate student behavior and attitudes (Rennie, 2003). Usually, in those studies, attitudes are surveyed with direct questions only (Kalichman & Friedman, 1992; Rennie & Rudland, 2003). The use of *explicit attitudes* is not unproblematic: due to the intrusiveness and threat of disclosure of such direct questions, the given answers by subjects may be biased by social desirability (Tourangeau & Yan, 2007; Bradburn et al., 2004).

Our paper adds to this literature by measuring *implicit associations* with the single-category implicit association test (SC-IAT) in a large-scale survey of scientists in Austria, Germany, and Switzerland. An IAT measures the relative strength of pairwise associations between concepts and attributes and has been frequently used in psychology to measure sensitive implicit attitudes (Greenwald et al., 1998). It has previously been applied to reveal information participants perceive to be sensitive and therefore might want to hide due to social desirability (Nosek et al., 2007). Until now, the IAT has mostly been applied to study implicit attitudes between “objects”, such as ethnic groups, products, spiders, etc. with corresponding attitudes, e.g. stereotypes, gender roles, or fear (see Hofmann, Gawronski, Gschwendner, Le, & Schmitt,^2005^,for an overview).

Here, we study the extent to which implicit attitudes among researchers connect specific behaviors (QRP and FFP) with academic success. As noted above, reporting forms of scientific misbehavior can be a difficult, sometimes career-ending decision among scientists, and is therefore certainly a sensitive question. The IAT thus helps us to better understand and quantify the connection between scientists’ associations towards scientific misconduct and its relation to perceived academic success. The underlying assumption we make is that some researchers consciously break rules of good scientific practice (e.g., falsify data or publish the same article twice), because they want to gain an advantage in the competitive scientific system, and the immediate returns from doing so outweigh the prospective sanctions of being caught.

Our data allows us to explore how socio-demographic factors connect to implicit attitudes. With a sample size of $N = 11,747$, conclusions can be drawn about differences in attitudes among specific subgroups, including gender, status, and academic field. Careful considerations can also be made about the country of academic training.

Our main finding is that a considerable fraction of about a fifth of researchers associate success with problematic research practices, while only a small minority connect FFP and success. Since attitudes are an important building block of later behavior, this may lead the way to misconduct and shows that more people may be engaging in it than those reporting it in surveys.

## Measuring implicit attitudes with the implicit association test

Attitudes are the psychological tendency to evaluate a certain (broadly understood) object with a certain degree of affection or aversion (Eagly & Chaiken, 1993; Fazio, 1990). These evaluations inherently possess a valence, encompassing positivity, negativity, or neutrality, and are universally applicable, extending for instance to behaviors, individuals, or social groups.

Since attitudes are mental states, they are inherently unobservable. Their measurement requires specific scientific tools, the most prominent of which are different forms of interviews, e.g. surveys or in-depth interviews. These measures are particularly suited to capture *explicit attitudes*. Explicit attitudes are conscious, we are able and sometimes willing to talk about them and can change them through active deliberation (Martinussen et al., 2018). They shape planned behaviors in deliberate situations.

*Implicit associations*, on the other hand, are “introspectively unidentified (or inaccurately identified) traces of past experience” (Greenwald & Banaji, 1995, p. 5). They are mostly inaccessible through deliberate communication, both to the person holding them as well as to the researcher (Nosek, 2007). Correlations between implicit and explicit attitudes exhibit considerable variability depending on the specific attitude (Nosek, 2007).

Implicit attitudes must be inferred through indirect, performance-based methods that bypass direct deliberative processing (Hahn et al., 2014). The Implicit Association Test (IAT) aims to measure the evaluative associations that underlie implicit attitudes by assessing the relative strength of pairwise associations between concepts and attributes. Widely utilized in psychology, particularly for gauging sensitive implicit attitudes, such as racism, the IAT has been employed to unveil information that participants might be inclined to conceal due to social desirability, given its indirect nature as a measurement of attitudes (Greenwald et al., 1998; Nosek, 2007).

The IAT evaluates the strength of associations between concepts and attributes (Greenwald et al., 1998). It employs a timed categorization task, wherein participants are presented with various stimuli, usually in pairs, and are instructed to rapidly categorize them based on pre-defined attributes (e.g., positive or negative). The test measures the speed and accuracy of these categorizations, with quicker responses indicating stronger associations. By tapping into automatic cognitive processes, the IAT aims to reveal unconscious biases and implicit attitudes that may not be easily accessible through explicit self-report measures. The IAT is based on the assumption that participants will exhibit quicker classification of a stimulus when the two concepts sharing the same response key are associated in their implicit attitudes, as opposed to when the two concepts sharing the same response key lack such implicit associations.

Meta-analyses examining the predictive validity of the IAT on behavior and explicit attitudes yield mixed results. In a meta-analysis by Hofmann et al. (2005), the correlation between the IAT and explicit self-report measures was examined. The findings indicate a general association between implicit and explicit measures. Similarly, in a meta-analysis conducted by Oswald et al. (2013), the predictive validity of the IAT and explicit measures of bias were assessed across various criterion measures of discrimination. The IAT demonstrated poor predictive ability for nearly all criterion categories, and its performance was comparable to simple explicit measures.

Greenwald et al. (2009) conducted a review of research reports assessing the predictive validity of Implicit Association Test (IAT) measures for behavioral, judgmental, and physiological outcomes. Concerning samples involving Black–White interracial behavior, IAT measures demonstrated significantly higher predictive validity than self-report measures. Both IAT and self-report measures exhibited incremental validity, contributing to the prediction of criterion variance beyond each other. Kurdi et al. (2019) conducted a meta-analysis exploring the conditions under which IATs measuring attitudes, stereotypes, and identity correlate with criterion measures of intergroup behavior, such as the behavior of white individuals towards black individuals. Implicit measures demonstrated predictive validity for all target groups and behavior types, unlike explicit measures.

## Data and methods

Our data come from a large-scale online survey (Zurich Survey of Academics, ZSoA) of scientists working in higher education institutions in Germany, Austria, and Switzerland in three different languages to choose from (German, English, French, Rauhut et al., 2024). The survey invitations were distributed to all Austrian and Swiss scientists working at universities and universities of applied sciences, as well as a randomly selected half of German scientists working at universities via email (see for detailed information Rauhut, Johann, Jerke, Rathmann, & Velicu 2021, p.38). The study was conducted between February and April 2020. All in all, 15,778 of nearly 141,000 scientists from 263 institutions answered, producing a response rate of approximately 11% (Rauhut et al., 2024, 2021). A majority of respondents (56%) are male, 42% are predoctoral researchers, 38% postdoctoral researchers, and 21% professors. Research fields include arts and humanities (47.9%), engineering (13.3%), life sciences (19.0%), and natural sciences (19.6%).

**Table 1:** IAT blocks

Block	Left	Right	Trials
Training block	Success	Failure	20
Testing block 1	Success & Behaviour	Failure	48
Testing block 2	Success	Failure & Behaviour	48

The order of the testing blocks was not randomized

The ZSoA included two different Single-Category Implicit Association Tests (SC-IAT), one covering questionable research practices (QRP) and the other covering scientific misconduct (FFP). The SC-IAT differs from the regular IAT by focusing on a single category instead of assessing associations between two contrasting categories (Karpinski & Steinman, 2006).^2^ In the SC-IAT, participants respond to stimuli associated with one category, and the test measures the strength of their implicit associations between that category and attribute concepts. This modification enables the investigation of biases or preferences related to a specific category without the traditional binary categorization used in the standard IAT. It offers a more targeted assessment of implicit attitudes within a singular context.

**Fig. 1:**
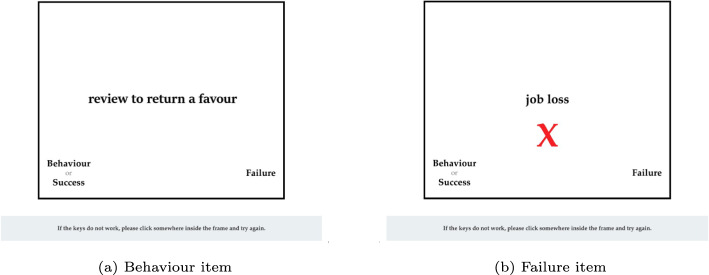
Screenshots of the IAT

Each participant was invited randomly to one of the two IATs consisting of three blocks each (see Table 1 for an overview of the different blocks). In the first block, a pure training block, the participants had to assign words for scientific success and failure to the attributes of success and failure. This block consisted of 20 trials. In the following two test blocks, the concepts of scientific misconduct were added, either QRP or FFP. These blocks each contained 48 trials. Between the test blocks, scientific behaviors alternated between failure on the left and success on the right. Figure 1 illustrates example images of the IAT. Further and more in depth methodological details can be found in the methods report of the survey (Rauhut et al., 2021).

Table 2 lists items corresponding to the respective attributes and concepts. The study included two tests: a QRP-IAT and an FFP-IAT, each with distinct items. QRP lacks an all-encompassing definition (for examples, see Fanelli, 2009), and can include many behaviors. However, because an IAT can only test a few concepts, we decided to use the items *honorary authorship*, *self-plagiarism*, *positive* * review to return a favour*, and *citation to return a favour*. For the FFP items, we followed the definition of The Office of Research Integrity (2018) and included *data falsification*, *data fabrication*, and *plagiarism*. We have also expanded the category to include *hiding conflicts of interest*, as these can also lead to the retraction of a scientific article (Gewinner et al., 2022) and undermine the integrity of the research process (Washington, 1992). For the valence attributes, science-specific attributes were chosen instead of general attributes such as “beautiful” or “good” to better integrate the IAT into scientific research. Opposite items were consistently used for positive and negative attributes.

**Table 2:** IAT items

Behaviour FFP	Behaviour QRP	Success	Failure
data falsification	honorary authorship	grant approval	grant rejection
data fabrication	self-plagiarism	publication success	publication failure
plagiarism	evaluation review to return a favour	high prestige	low prestige
hide conflicts of interest	citation to return a favour	reputation	lose face
		tenure	job loss

Participants were shown either QRP or FFP items together with success and failure items

See Table A1 for translations and the percentage of responses in each language

## Measures

Our main measure of implicit attitudes is the IAT score based on the *improved algorithm* introduced by Greenwald et al. (2003). This algorithm is based on Greenwald’s (1998) original measure but includes defined criteria for excluding excessively slow participants. It calculates the difference between the time it takes a respondent to associate a misconduct behavior with a positive word (success) and the time to connect the same behavior to a negative word (failure). Faster answers indicate stronger associations, which indicates a stronger bias which means the quicker someone answers the more true they answer to their implicit bias.

The improved algorithm exhibits superior performance in correlations with self-reports, resilience against response speed artifacts, internal consistency, sensitivity to known influences on IAT measures, and resistance to procedural influences (Greenwald et al., 2003).

Our dataset contains data from 13,532 respondents that actually start the IAT and from each trial of all test blocks we administered. Since mitigating the influence of self-presentation and controlled responses is an important objective of the IAT (Fiedler & Bluemke, 2005), we initially excluded trials with response times exceeding 10,000ms to clean the data. Simultaneously, participants with latencies exceeding 300ms in more than 10% of the trials were removed from the sample. As a consequence, an IAT is hardly fakeable by the respondents (Fiedler & Bluemke, 2005; Schnabel et al., 2008), as controlled, and therefore not implicit, responses and participants get dropped from the dataset.

Next, we calculated the average latency for each participant in every block, thus the average time to press the correct key within each block. Secondly, we calculated a combined standard deviation over both testing blocks. Following Greenwald et al. (2003), in instances where latency data were unavailable, such as due to transmission errors, the procedure prescribes that the mean latency has to be inserted. However, this situation did not occur in our case as all was correctly transmitted.

Finally, the mean latency was determined for each block per participant, and the difference between testing block 1 and testing block 2 was computed. This difference was then divided by the corresponding pooled standard deviation. Subsequently, the mean value derived from both results constituted the IAT score. A negative value signifies the implicit association of QRP/FFP with failure, while a positive value indicates the implicit association of QRP/FFP with success. Scores falling within the range of $-0.15$ to 0.15 are interpreted as indicative of no preference. Scores between 0.16 and 0.35 correspond to slight preferences, and 0.36 to 0.65 correspond to moderate preferences, respectively. Values exceeding 0.65 in absolute magnitude are considered a strong preference (Hahn et al., 2014). These classifications also follow the conventions of Project Implicit (2011). Although some scientists criticize these classifications as arbitrary (e.g., Blanton & Jaccard, 2006), Greenwald et al. (2006) defend their use by pointing out that they are based on commonly used classifications in science for interpreting effect sizes.

Since latency, i.e., the time interval between the on-screen display and the respondent’s keyboard input is fundamental for calculating the IAT scores, the survey underwent preliminary testing on contemporary browsers—Google Chrome, Microsoft Internet Explorer, Mozilla Firefox, and Apple Safari. Participants attempting to access the survey on a mobile device with a mobile operating system were advised, at the survey’s start, to use a desktop or laptop PC for optimal completion. The Implicit Association Test (IAT) was implemented using Javascript on the client side. Latencies were cached and only sent to the server once the test was completed. This ensured that a poor internet connection did not affect the latency times.

In addition to the IAT-Score, we collected a wide range of other measures and socio-demographic information (see Table A3). In the following, we will concentrate on academic status (pre-doctoral, post-doctoral, and professors), gender (male and female), scientific discipline (humanities, natural sciences, life sciences, and engineering), as well as the world region from which the respondent has obtained his or her PhD (America, Asia, or Europe). Africa and Oceania had to be excluded because we had very few cases from these regions (see Table A2). Pre-doctoral respondents are categorized as obtaining it from the country of their current institution, i.e., Austria, Germany, or Switzerland.

## Results

**Fig. 2:**
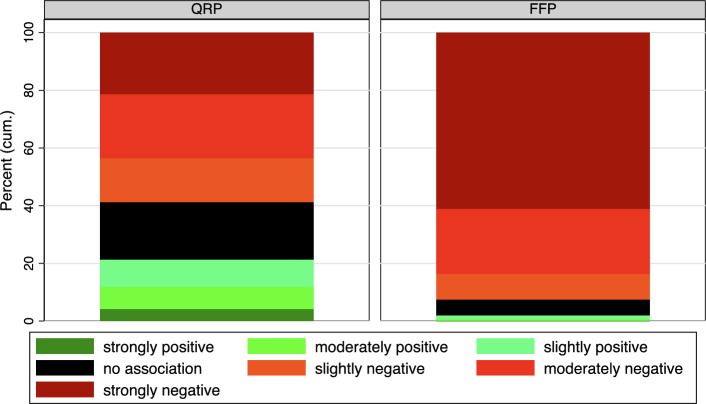
Distribution of IAT Scores. See Table A4 in the Appendix for exact percentage values

The following figures are presented as discrete distributions for illustrative purposes, although the underlying data is metric. Figure 2 illustrates the distribution of IAT scores concerning QRP on the left and FFP on the right. The color-coded bars represent the categorized IAT scores. A significant portion of participants implicitly associate QRP with failure, with approximately 20% exhibiting no clear association with either success or failure. Notably, a considerable proportion of respondents demonstrate positive associations between QRP and success. In contrast, the results for FFP indicate lower associations between scientific misconduct and success. The T-test confirms significantly higher associations between QRP and success (T-test, $t(11745)=56.7$ $p<0.001$).^3^ It is also noteworthy that there are only a few respondents who display no clear preference. In addition, we performed an F-test to check whether QRP and FFP, finding no evidence for that (F-Test, $F(2,11745)=3215$ $p < 0.001$), suggesting significantly higher associations between QRP and success as compared to FFP and success. This analysis also serves as a robustness test of the IAT, as we expected respondents to have less negative implicit attitudes toward QRP than toward FFP, comparable to attitudes toward minor and major crimes (Cullen et al, 2019).

**Fig. 3:**
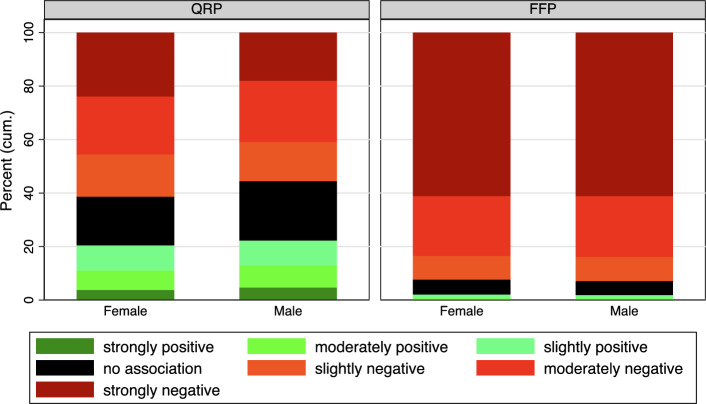
Categorized IAT Score by Gender. See Table A5 in the Appendix for exact percentage values

Figure 3 depicts the IAT scores for QRP and FFP categorized by the gender of the respondents. Overall, there are minimal, if any, discernible differences between the genders. On average, women have a slightly higher implicit association of QRP with success compared to men (female = 3.0, male = 3.2, T-test, $t(5833)=4.6$ $p<0.001$). When examining the IAT scores related to FFP, no gender differences are evident (female = 1.66, male = 1.65, T-test, $t(5910)=0.46$ $p>0.05$). In order to be able to determine a difference between the genders in the IAT scores, we also carried out an F-test for the QRP and FFP scores. There is evidence that the distribution of QRP scores differs significantly between men and women (F-Test, $F(1,5833)=21.7$ $p<0.001$), with women on average appearing to be more likely to implicitly associate QRP with success. However, the difference in averages is rather small, suggesting a slightly higher variance among women’s IAT-score. For FFP, we find no significant differences in the distributions between men and women (F-Test, $F(1,5910)=0.22$ $p>0.05$)

**Fig. 4:**
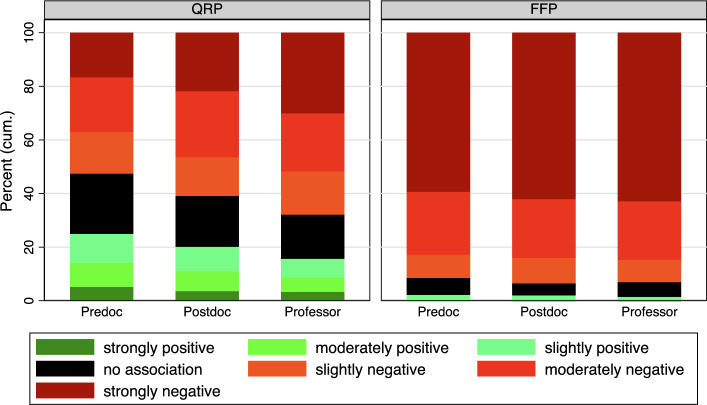
Categorized IAT Score by Academic Status. See Table A5 in the Appendix for exact percentage values

When considering academic status, substantial differences become apparent (Figure 4): A distinct status effect is observed in the implicit associations with QRP (F-test, $F(3, 5832)=53.1$ $p<0.001$). PhD students tend to associate QRP with success significantly more frequently than post-docs (Predoc = 3.4, Postdoc = 3.1, T-test, $t(4635)= 6.38$ $p<0.001$), while the proportion among professors is even lower than that of post-docs (Postdoc = 3.1, Professor = 2.8 T-test, $t(3371)= 4.6$ $p<0.001$). Moreover, the percentage of respondents that associate QRP with neither success nor failure decreases with increasing status. We find smaller but existing differences in the association of FFP between Predocs and the other groups (T-test, Predoc = 1.69, Postdoc = 1.63, $t(4745)=2.0$ $p<0.05$, Postdoc = 1.63, Professor = 1.61 $t(3413)=0.42$ $p>0.05$). However, the graphical analysis shows that this is particularly pronounced for QRP, while the differences for FFP are very small.

**Fig. 5:**
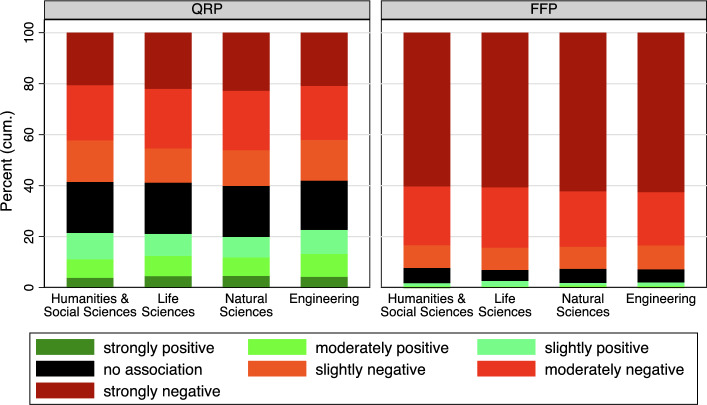
Categorized IAT Score by Discipline. See Table A4 in the Appendix for exact percentage values

In Figure 5, respondents were categorized into broad subject groups—humanities, natural sciences, life sciences, and engineering. Minor distinctions that are not statistically significant exist among the subject groups: Engineering exhibits the highest proportion of respondents associating QRP with success, while the natural sciences show the smallest proportion, the humanities and life sciences fall in between (humanities = 3.15, life sciences = 3.11, natural sciences = 3.07, engineering 3.19, F-test, $F(3,5831)=0.94$ $p>0.05$). When examining associations between FFP and success, once again, no differences are discernible among the subject groups (humanities = 1.66, life sciences = 1.65, natural sciences = 1.64, engineering 1.64, F-test, $F(3,5908)=0.2$ $p>0.05$).

**Fig. 6:**
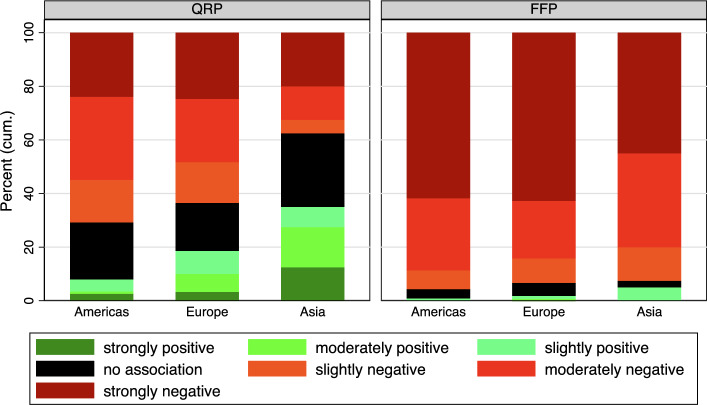
Categorized IAT Score by world region of PhD-granting university. Current PhD are excluded in this graph. See Table A5 in the Appendix for exact percentage values

Lastly, we examine differences in associations between QRP and FFP concerning *academic origin* (Figure 6). Academic origin refers to the country where the respondent obtained their doctorate, thus limiting the sample to respondents of at least postdoc-status. Respondents still in the process of completing their doctorate during the survey were categorized under Europe. Due to the very limited number of respondents who completed their doctorate in Africa or Oceania, these regions were excluded.^4^ It is important to note that all the regions depicted are predominantly influenced by a few countries: The USA dominates the Americas, and China and India dominate Asia due to their large size. Europe is mainly influenced by Germany, Austria, and Switzerland, as the ZSoA surveyed scientists from these countries.

Substantial differences emerge among the three academic regions of origin presented: Individuals who completed their doctorate in Asia are substantially more inclined to associate QRP with success than those with a doctorate from Europe or the Americas (Asia = 3.85, Americas = 2.64, Europe = 2.95, F-test, $F(2,3297)=7.5$ $p<0.001$). Simultaneously, the proportion of respondents expressing indifference is also notably higher. In comparison to individuals pursuing or having completed a doctorate in Europe, the percentage of individuals with a doctorate from the Americas and a positive association with QRP is significantly lower.

Turning attention to FFP, the differences are considerably smaller: The proportion of positive associations is roughly similar across all regions but at a very low level (Asia = 1.87, Americas = 1.55, Europe = 1.62, F-test, $F(2,3332)=21$ $p>0.05$). However, it is noteworthy that the percentage of indifferent individuals is higher among respondents who underwent academic socialization in Asia compared to the Americas or Europe.

As a last step, we estimate a multiple linear regression to control for the respective other explanatory variables presented above. This allows us to check the robustness of our results with respect to, e.g., over-representation of women in early career stages or German-speaking respondents in the humanities and social sciences. Table 3 presents four regressions, two for QRP and two for FFP. All specifications show only marginal and insignificant associations between disciplinary background and associations with misbehavior. Model 1 shows a significantly more positive association between QRP and female respondents, also controlling for academic status. This association also holds when looking at mid- to advanced career researchers and controlling for the region where the respondent received their PhD (Model 2). However, we find no differences when looking at the relationship between gender and associations towards FFP (Models 3 and 4). Finally, the negative association between academic status and positive associations towards QRP and FFP also holds in the multiple regressions.

**Table 3:** Multiple linear regression models explaining positive associations towards Questionable Research Practices (QRP, columns 1–2) and Falsification, Fabrication and Plagiarism (FFP, column 3–4)

	(1)	(2)	(3)	(4)
	QRP	QRP	FFP	FFP
Humanities & Social Sciences	Ref.	Ref.	Ref.	Ref.
Life Sciences	−0.0107	−0.0303	−0.00127	−0.0113
	(−0.60)	(−1.33)	(−0.09)	(−0.61)
Natural Sciences	−0.00798	−0.0327	−0.00320	−0.00680
	(−0.44)	(−1.39)	(−0.22)	(−0.35)
Engineering Sciences	−0.00261	−0.00638	−0.0132	0.0284
	(−0.13)	(−0.21)	(−0.82)	(1.18)
Female	0.0463***	0.0568**	−0.00471	0.00572
	(3.34)	(3.05)	(−0.42)	(0.38)
Predoc	Ref.	N.a.	ref.	N.a.
Postdoc	−0.0991***	Ref.	−0.0274*	Ref.
	(−6.65)		(−2.28)	
Professor	−0.173***	−0.0763***	−0.0515***	−0.0254
	(−9.69)	(−4.08)	(−3.50)	(−1.66)
Europe		ref.		Ref.
Americas		−0.0405		0.00453
		(−0.84)		(0.12)
Asia		0.235**		0.103
		(2.94)		(1.58)
Constant	−0.190***	−0.286***	−0.717***	−0.752***
	(−12.47)	(−15.95)	(−58.45)	(−52.93)
N	5835	3300	5912	3333
$R^2$	0.0221	0.0129	0.00226	0.00236

*t* statistics in parentheses

* $$p<0.05$$, ** $$p<0.01$$, *** $$p<0.001$$

QRP (1) and FFP (3) include all scientists.QRP (2) and FFP (4) add PhD country information

The sample size is therefor restricted to postdocs and professors

## Discussion

In summary, our application of the Implicit Association Test (IAT) revealed that there is unlikely to be an association between serious scientific misconduct and success. However, for questionable research practices (QRP), we cannot make the same confident assertion. Approximately one-fifth of the participants implicitly associate QRP with success, at least to some degree. No differences were found in the implicit attitudes towards QRP and FFP among different subject groups. Additionally, there were only minor differences between men and women in their implicit associations with QRP. While there is some evidence that women are more likely to associate QRP with success, this effect is not observed with FFP. It is worth noting that PhD students, and to a lesser extent post-doctoral researchers, are more likely than professors to associate QRP with success. A similar effect is found regarding FFP, although it should be noted that the effect of FFP is barely noticeable. Furthermore, differences in associations are found based on academic origin, specifically the region in which one obtained their Ph.D. Participants who were socialized in Asia are more likely to associate QRP with success than those who were socialized in Europe or the Americas.

The association between QRP and success decreases with rising status, which may seem counterintuitive. As individuals gain more experience or age, they tend to adhere more closely to established standards, or the relationship between explicit expectations and implicit social desirability weakens over time. However, it is unclear whether this is a cohort effect, indicating a broader cultural or systemic shift in scientific practices and ethical considerations, which would be less beneficial to the scientific community. Nevertheless, it is unclear whether a concept’s implicit association, whether positive or negative, such as racism or QRP and FFP in our case, actually predicts certain behavior. Meta-analyses that examine the predictive validity of the IAT on behavior and explicit attitudes yield mixed results (e.g., Greenwald et al., 2009; Oswald et al.,^2013^). Some analyses find that IAT scores are equal to explicit measures in predicting behavior, while others find that implicit measures better predict behavior.

If the observed effect is related to status, it implies a need to focus on training efforts for younger personnel. Early intervention, possibly emphasizing ethical concerns in science during undergraduate and graduate degrees, could help shape young researchers’ perspectives on problematic research procedures and norms. It is critical to address the potential cohort effect to understand these results and direct future research efforts in this field. It is noteworthy that vulnerable groups, such as women compared to men, doctoral students compared to more experienced academics, and individuals who have immigrated to the Germanophone academic system from non-Western countries compared to academics from Europe or the Americas, exhibit more positive implicit associations with QRP.

Another interpretation of our findings is related to success. Vulnerable groups may feel insecure in their careers due to systemic biases and challenges they face in the academic environment. These challenges can include limited access to resources, mentorship, and networking opportunities, as well as experiences of discrimination and bias. This insecurity might lead them to view academic success as inherently unfair, as they may perceive that it is often achieved through unequal opportunities rather than merit. Consequently, they might associate success with QRP.

It is important to note that this study has limitations. The IAT used in this study lacks validation against explicit attitudes or behavioral measures and other implicit assessments, underscoring the need for future research in this direction. One interesting approach would be to conduct a validation study with known misconduct perpetrators and compare their scores on a misconduct IAT to those of scientists without a history of scientific misconduct. However, such a study would be very difficult to set up.

Validation against other explicit measures can be challenging. According to Fazio et al. (1995), substantial correlations between implicit and explicit measures are expected in neutral domains. However, in socially sensitive subjects, null or even negative correlations are expected. Given that QRP and FFP squarely fall within the realm of socially sensitive topics, validating them through assessing the correlation of explicit and implicit attitudes toward scientific misconduct appears to be a complex endeavor.

Moreover, individual concepts may have different connotations in different cultural contexts as well as in different languages. For instance, “honorary authorship” may have invoked different ideas for different respondents, and that respondents not native in either of our three survey languages may even have had even further associations. That said, it is nothing that can be completely avoided when running surveys in a cross-cultural setting, and we did our best to avoid misleading translations in the survey. We would like to point out that this effect might be mitigated by the fact that the IAT was at the end of a big survey asking participants about different aspects of academic life, and in particular about different kinds of QRPs and FFPs. The concepts behind the practices used in the survey should therefore be familiar to the respondents and reasonably similarly explained, such that cultural/linguistic particularities should play a lesser role than if respondents had been confronted with the terms out of thin air.

The initial results of the analysis of our two IATs serve as a proof-of-concept that can guide further research into questionable scientific behavior and scientific misconduct. It was easy to implement within the ZSoA, and respondents could answer it quickly. The IAT is an established measure in psychology, particularly in relation to sensitive topics such as racial and gender bias, making it a valuable addition to the science research toolbox. Future research is needed to determine whether the IAT can be a useful tool for measuring attitudes in the context of science studies.

## Supplementary Information

Below is the link to the electronic supplementary material.

Supplementary file1 (PDF 13 kb)

## Footnotes

1 Similar definitions exist for the EU (Drenth, 2012) China (The General Offices of the Communist Party of China Central Committee and the State Council, 2019) and other world regions.

2 In this text, we will only use the term IATs when referring to the association tests for the sake of clarity. However, this always pertains to the single-category IAT that we utilized in this project unless otherwise specified.

3 Replication code is available at https://doi.org/10.17605/OSF.IO/XTESF

4 The comprehensive figure is available in the Supplemental Material as Figure A2, accompanied by a table detailing the number of respondents from each region (Table A3).
