# Supplementary material for: Are questionable research practices considered a successful career strategy? A novel implementation of the implicit association test
Source: Scientometrics. 2025 Jul 5;130(7):3367–82. doi: 10.1007/s11192-025-05357-4 (PMC12374866; doi:10.1007/s11192-025-05357-4)
Supplement: Supplementary file 1 — Supplementary file1 (PDF 13 kb) [file 11192_2025_5357_MOESM1_ESM.pdf]

Supplementary Information. The IAT was located at the end of the survey, the transition took place with the information: *"You will now be redirected to the second varied part and hence to the end of the survey. If you click on "Next", you cannot make any changes to the previous answers."* The instruction of the IAT was: *"In the following task we ask you to assign words to the correct categories as fast as possible. This task will take about 5 minutes. Following is a list of categories and the words that belong to each category:"* and the terms are listed in Table A1.

Table A1: Categories and terms of the IAT in ZSoA translated in the three languages.

Category	German (76% of respondents)	English (17.4% of respondents)	French (6.6% of respondents)
Success	Antragsbewilligung, Publikationserfolg, Guter Ruf, Reputationsgewinn, Festanstellung	grant approval, publication success, high prestige, reputation gain, tenure	demande accept´ee, succ`es de publication, bonne r´eputation, b´en´efice de r´eputation, poste fixe
Failure	Antragsablehnung, Publikationsmisserfolg, Schlechter Ruf, Gesichtsverlust, Jobverlust	grant rejection, publication failure, low prestige, loss of face, job loss	demande rejet´ee, ´echec de publication, mauvaise r´eputation, perdre la face, perte d'emploi
QRP behaviour	Ehrenautorschaft, Selbstplagiat, Gefa¨lligkeitsgutachten, Gefa¨lligkeitszitat	honour authorship, selfplagiarism, review to return a favour, citation to return a favour	auteur·e honoraire, auto-plagiat, rapport de complaisance, citation de complaisance
FFP behaviour	Datenfa¨lschung, Datenmanipulation, Plagiat, Interessenkonflikt verschweigen	data falsification, data manipulation, plagiarism, conceal conflict of interst	falsification de donn´ees, manipulation de donn´ees, plagiat, dissimulation de conflits d'int´er^ets

Figure A1 illustrates the density distribution of IAT scores, with the left side representing QRP items and the right side representing FFP items. The background color indicates the categorized strength of association (refer to section *Data & Methods*). The *x*-axis quantifies the strength of the implicit association with success or failure for QRP and FFP, where smaller values indicate a strong association with failure, while larger values indicate a stronger association with success.

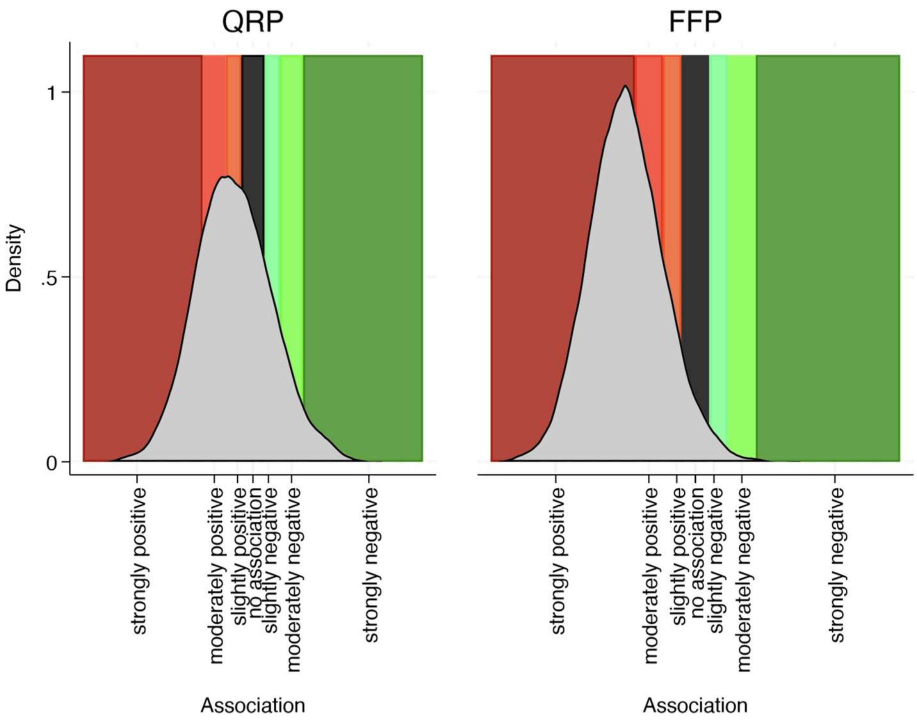

Fig. A1: Distribution of IAT Scores

Table A2 displays the distribution of observations across different regions where participants completed their doctorates. China and India dominate the Asia region, while the USA is the predominant contributor in the Americas. Individuals who have not yet completed their doctorates were categorized under Europe.

Table A2: Number of Observations by Region

Region	IAT QRP	IAT FFP	Total
Americas	113	115	228
Europe	3,147	3,178	6,325
Asia	40	40	80
Africa/Oceania	13	17	30

Unknown/Undefined	2,522	2,562	5,084
Total	5,835	5,912	11,747

Table A3 shows the descriptive statistics for key variables, including gender, academic status, discipline and world region of PhD.

Figure A2 displays the IAT scores categorized by the regions where participants completed their doctorates. This graph represents the complete version of Figure 6. In the text version, Africa and Oceania were omitted due to the limited number of observations in these regions ($N_{\text{Africa/Oceania}} = 30$). A Kruskal-Wallis rank sum test, including all regions, shows significant differences in the distribution of QRP scores (Kruskal-Wallis $\chi^2(2) = 8.57$ $p < 0.05$), but no differences in the distribution of FFP scores (Kruskal-Wallis $\chi^2(2) = 3.95$ $p = 0.267$).

Table A4 presents the distribution of observations based on categorized IAT scores. Notably, about 60% of participants show an implicit association between QRP and failure, with this percentage soaring to over 90% for FFP. Conversely, roughly 17% of participants display no implicit association between QRP and either failure or success, while this figure drops to approximately 5% for FFP. Intriguingly, around 20% of respondents associate QRP with success, in stark contrast to the less than 1% who link FFP with success. It further shows the associations by discipline, and language of the questionnaire. Table A5 presents the distribution of observations by gender, academic status and world region of PhD-granting institution.

Table A3: Descriptive Statistics.

A detailed analysis of the representativity of the sample compared to the overall population of scientists in Germany, Austria, and Switzerland can be found in the methods report (Rauhut et al., 2021, p.35f).

	Summary
**N**	11,747
**Gender**	
Female	6,632 (56.5%)
Male	5,115 (43.5%)
**Age**	
<30	2,598 (22.1%)
30-39	4,841 (41.2%)
40-49	2,035 (17.3%)
50-59	1,626 (13.8%)
60+	646 (5.5%)
n.a.	1 (0.0%)
**Discipline**	
Humanities & Social Sciences	5,349 (45.5%)
Life Sciences	2,228 (19.0%)
Natural Sciences	2,282 (19.4%)
Engineering Sciences	1,888 (16.1%)
**Academic status**	
Predoc	4,959 (42.2%)
Postdoc	4,425 (37.7%)
Professor	2,363 (20.1%)
**Language of IAT**	
English	2,059 (17.5%)

French	776 (6.6%)
German	8,912 (75.9%)
**World region**	
Americas	228 (1.9%)
Europe	6,325 (53.8%)
Asia	80 (0.7%)
Africa/Oceania	
Unknown/Undefined	5,084 (43.3%)

* 30 (0.3%)

Africa and Oceania are excluded in the main analyses.

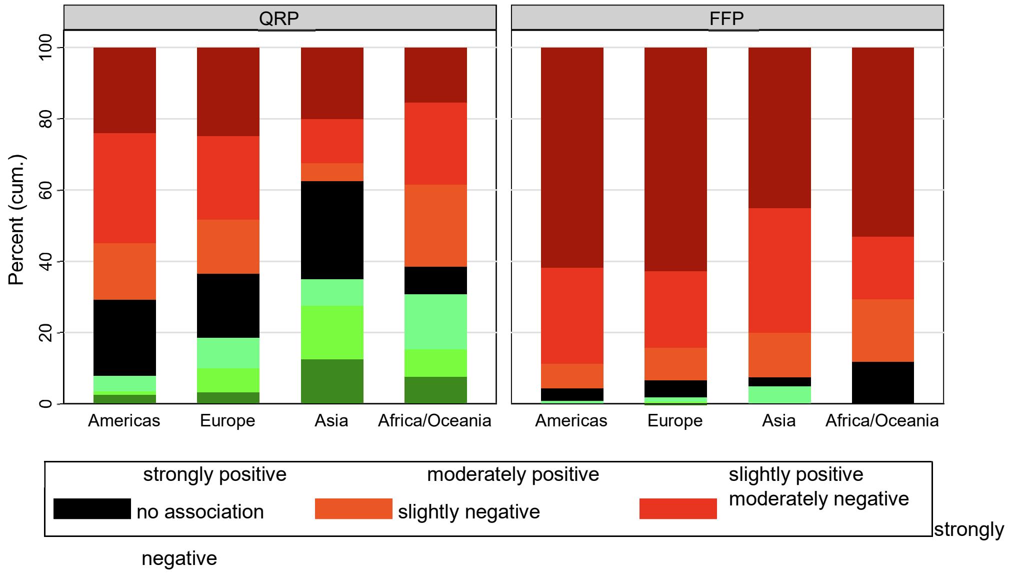

Fig. A2: Categorized IAT Score by PhD Region.

TableA4 AssociationstowardsQRPandFFPinthecompletedata,bydiscipline,andlanguageofthequestionnaire.

	CompleteData	Discipline	Language
		SocialLifeNatural SciencesSciencesSciencesEngineeringEnglishFrenchGerman	
		%12.5%0%	
	QRPFFP	QRPFFPQRPFFPQRPFFPQRPFFP	QRPFFPQRPFFPQRPFFP
stronglynegative	20.5%60.3%	%60.7%22.7%62.2%23.9%61.7%20%45%	20.9%62.5%22.3%55.8%24.7%62.7
		%0.0%4.4%0.0%7.5%5%	
moderatelynegative	21.7%23%	23.4%23.6%23.3%21.8%31%27%12.5%35	21.1%20.9%24.9%24%23.5%21.5
slightlynegative	16.3%8.9%		%9.3%14.9%10.6%15.2%9.1%
			%0.0%3.3%0.0%
noassociation	20%5.9%	20.1%4.3%20%5.6%21.2%3.5%27.5%2.5	19.3%5.2%19.1%6.5%17.9%4.9
slightlypositive	10.3%1.2%	13.4%8.8%14.1%8.6%15.9%7%5	9.4%1.3%8.9%2.2%8.6%1.1
moderatelypositive	7.3%0.0%	%0.0%7.3%0.0%0.0%0%15%0%	%0.0%6.9%0.0%6.7%0.0%
stronglypositive	3.9%0%		
Total	100%100%	100%100%100%100%100%100%100%100	100%100%100%100%100%100

%

%

%

%

%

%

%

%

4.5%0.0%4.5%0.0%2.7%0

16 4.3%0.0%3

22 8.6%2.2%8

9

8

Table A5 Associations towards QRP and FFP by gender, academic status and world region of PhD-granting institution

	Gender				Academic Status						Region					
	Male		Female		Predoc		Postdoc		Professor		Asia		Europe		Americas	
	QRP	FFP	QRP	FFP	QRP	FFP	QRP	FFP	QRP	FFP	QRP	FFP	QRP	FFP	QRP	FFP
strongly negative	29.4%	59.1%	20.4%	62.5%	[illegible]	61.1%	23.9%	61.1%	16.7%	59.4%	21.7%	62.1%	30.1%	62.9%	21.3%	61.1
moderately negative	23.9%	21.7%	21.5%	22.3%	22.9%	22.7%	21.7%	22.4%	20.3%	23.5	24.6%	22%	21.7%	21.8%	22.2%	22.6
slightly negative	9.4%	9.7%	15.9%	8.5%	[illegible]	15.8%	8.8%	15.6%	8.7%	[illegible]	14.5%	9.5%	16.1%	8.3%	15.3%	8.9
no association	18.2%	5.9%	20.3%	5.2%	22.2%	5.3%	18.2%	5.6%	22.5%	6.3	18.9%	4.5%	16.4%	5.5%	19.9%	5.4
slightly positive	7.8%	1.8%	9.6%	1.1%	9.3%	1.2%	9.5%	1.4%	10.8%	1.6	9.2%	1.3%	6.9%	0.0%	9.4%	1.3
moderately positive	7.5%	1.8%	7.9%	0.0%	8.3%	0.0%	7.2%	0.0%	9.1%	0.0	7.4%	0.0%	5.4%	0.0%	7.7%	0.0
strongly positive	3.9%	0%	4.5%	0.0%	4.7%	0.0%	3.8%	0.0%	5.2%	0.0	3.6%	0.0%	3.3%	0.0%	4.2%	0.0
Total	100%	100%	100%	100%	100%	100%	100%	100%	100%	100	100%	100%	100%	100%	100%	100

18 14.6%9
